# Reaching Out of the Box: Effective Emergency Care Requires Looking Outside the Emergency Department

**DOI:** 10.5811/westjem.2016.5.30244

**Published:** 2016-06-21

**Authors:** Daniel A. Dworkis, David A. Peak, Jason Ahn, Tony A. Joseph, Ed Bernstein, Eric S. Nadel

**Affiliations:** * Harvard Medical School, Department of Emergency Medicine, Boston, Massachusetts; † Boston University Medical Center, Department of Emergency Medicine, Boston Massachusetts

## INTRODUCTION

Patients do not start to exist when they arrive at the door of our emergency departments (ED), nor do they stop existing when they leave. Instead, before they fall ill or become injured they live and exist somewhere and when they are discharged from our care they will likely return to that same somewhere. As emergency providers (EPs), our attention must be focused on the patients in front of us, but fundamentally the details of this “somewhere” directly affect our ability to provide safe and effective emergency care. Specifically, both patient-specific factors like homelessness, immigration status, living situation, or insurance coverage, and structural factors arising from broader community and societal forces like food deserts, community violence, and poor housing quality can strongly impact both emergency presentations and our ability to safely and effectively discharge patients. Here, we argue that our duty as EPs extends beyond the four walls of our EDs into life in our communities, and that understanding and addressing the unique strengths and needs of the communities we serve is a crucial component of our ability to provide effective emergency care.

## WHERE DID YOU COME FROM?

A 45-year-old female patient presenting with a cough might raise different sets of concerns if she comes to the ED from her apartment, a homeless shelter, or Western Africa. Context and community obviously matter in terms of the pre-test probabilities assigned to potential diagnoses, and EPs need to be aware of the community-level risk factors they are likely to see. This connection is especially true for vulnerable populations such as homeless individuals whose social context might influence their potential exposures or ability to access care.^1^ However, the interaction between the details of the reality outside of the ED and acute emergency health needs runs deeper than simple adjustments of pre-test probability.

Consider, for example, if our patient’s cough is due to an exacerbation of her asthma; ED visits for asthma flares have been linked to outside-the-ED factors like socioeconomic status and local levels of ozone exposure.^2^ Difficulties obtaining the needed controller medications such as cost and variability in access to commercial pharmacies and affordable generic drugs might also play roles in a patient transitioning from a manageable degree of symptoms into an acute episode requiring emergency care.^3^ These effects are not limited to visits for asthma or other chronic disease states; outside of the ED factors such as race and insurance status have similarly been shown to be related to exposure to and survival after non-accidental trauma.^4^

As EPs, we often ask patients why they presented here and now with this specific complaint as opposed to presenting at a different time or place. Rarely do they respond with a multi-factorial analysis of relative levels of ozone exposure and driving distance to their local pharmacies, but the truth is that there is a densely connected network of social factors existing outside the walls of the ED that can directly impact our patient’s emergency needs. Significant amounts of mapping and analyses of these networks of factors have been performed in non-ED settings, most notably led by the World Health Organization’s (WHO) Social Determinants of Health Unit, and more work is needed to understand social factors at the patient and community levels that influence emergency care needs.^5^ To paraphrase Sir Michael Marmot, former chair of the WHO Commission on Social Determinates of Health, having an emergency may be a personal issue, but the rate of needing an ED is a societal issue.^6^

## WHERE ARE YOU GOING?

Continuing our example, our patient with an asthma flare has improved after treatment and we make a plan to discharge her home with a short course of steroids, refill her albuterol inhaler, and instruct her to see her primary care doctor in one week. Safely discharging patients back into their communities is a key skill for EPs; however, some discharges fail and patients may return for “bounce back” ED visits or otherwise suffer adverse health outcomes.

EPs may think of our discharge plans as perfect and an inability to follow through with it as a failure on the part of our patients. In reality, however, both patient-specific and structural factors originating outside the ED can make our discharge plans impractical if not impossible to execute. Poverty, hunger, and lack of insurance or underinsurance have all been shown to be related to patients’ probabilities of following through with ED discharge plans or even simply purchasing recommended medicines.^7^ In Boston, MA, work by our team and others has highlighted several patient-level and structural factors that can significantly impact the efficacy of discharges from our EDs; for example, homeless individuals with chronic lung disease were found to be largely unable to use their recommended maintenance or rescue medications in Boston-area homeless shelters due largely to a lack of electrical outlets in shelters.^8^

Within the Knowledge, Skills, and Abilities (KSAs) profiles set out by the American Board of Emergency Medicine (ABEM), KSA DI0 (“Disposition-0”) states that EPs should be able to “[e]stablish and implement a comprehensive disposition plan that uses appropriate consultation resources; patient education regarding diagnosis; treatment plan; medications; and time and location specific disposition instructions.”^9^ To accomplish this, EPs need to recognize groups of patients in the ED who are vulnerable for failing outpatient discharge based on the characteristics of their emergency presentation and course of ED treatment, as well as groups who might be unable to complete a discharge plan because of barriers they face outside of the ED. These barriers might be broad, such as hunger, health literacy, or insurance issues, or they might be unique to the microenvironment of a particular ED: for example, EDs discharging patients in Boston’s neighboring cities might find homeless shelters with sufficient electrical outlets but a host of different potential barriers that require understanding and potential intervention outside of the ED. Discharge instructions represent a plan to be carried out by a particular person in a particular community and if patients are to succeed at these plans, EPs need to understand the unique strengths and constraints of the communities they expect the plan to function in.

## WHERE CAN WE GO TOGETHER?

If visits to and discharges from EDs are significantly impacted by conditions outside of the ED, how should EPs begin to account for these conditions in the context of patient care? KSA MF0 (“Modifying Factors-0”) states that EPs should be able to “[a]djust treatment of patients according to factors such as culture, gender, age, language, disability, and social status;” however, it does not define “social status,” nor does it offer specifics on how that might influence our care.^7^ We believe that more work is needed to recognize and develop training and competencies addressing the social realities that shape our patients’ emergency needs. Toward that end, we would offer the following potential structure for improving the ways EPs and EDs respond to the needs of their communities.

First, all EPs should be able to understand and identify key factors at the patient-specific and structural level that might influence a patient’s presentation or discharge plan. This would include an improved screening system using validated tools to identify social determinants of health, as well as a more in-depth understanding of the broader forces at work in the community served by the ED. Implicit in this idea are the assumptions that (1) each community has a different profile of risks and strengths much like each patient does, that (2) EPs will need to actually leave the ED (themselves or by proxy) in order to understand how their community actually works, and that (3) these factors are likely to change over time and EPs will need to maintain open communication with their communities to identify new and changing barriers to care.

Second, once EPs have identified social factors, we should use, where available, pre-existing resources that are designed to address these factors. These might include social workers or case managers already embedded in the ED, or referrals to programs outside of the ED like food pantries, free clinics, or programs like the Boston-based Breathe Easy at Home program, which conducts home visits for children with asthma to assess for sub-standard housing conditions that might contribute to asthma flares and then provides legal support for changing these conditions.^10^ Using this type of resource, EPs could direct further resources outside the ED to particular patients within their community that they identify during their work inside the ED.

Finally, EPs with particular interest in the social determinates of their patients’ health could go even further and work to develop new ED resources tailored to address these factors, for example, centers of research like the Oakland, CA-based Andrew Levitt Center for Social Emergency Medicine, or peer-education based programs like Boston-based Project ASSERT.^11^,^12^

In order to accomplish these goals, we as EPs need to make thinking outside the walls of our ED a new priority: while the core of our specialty remains the provision of the highest quality emergency medical care to all who are in need of it, we must recognize that our ability to provide this care is directly linked to our ability to deeply understand the reality of the lives of our patients and our communities.
